# Enhancement of Gaseous *o*-Xylene Elimination by Chlorosulfonic Acid-Modified H-Zeolite Socony Mobil-5

**DOI:** 10.3390/molecules29153507

**Published:** 2024-07-26

**Authors:** Yaxu Wang, Xiaolong Ma, Hongmei Wang, Dandan Zhao, Yuheng Liu, Zichuan Ma

**Affiliations:** 1 Hebei Key Laboratory of Inorganic Nano-Materials, College of Chemistry and Material Sciences, Hebei Normal University, Shijiazhuang 050024, China; yaxuwang@stu.hebtu.edu.cn (Y.W.); wanghm@stu.hebtu.edu.cn (H.W.); zhaodd@stu.hebtu.edu.cn (D.Z.); 2 School of Environmental Science and Engineering, Hebei University of Science and Technology, Shijiazhuang 050018, China; maxiaolong2410@hebust.edu.cn; 3 Hebei Key Laboratory of Innovative Drug Research and Evaluation, College of Pharmaceutical Sciences, Hebei Medical University, Shijiazhuang 050017, China

**Keywords:** HZSM-5, chlorosulfonic acid modification, *o*-xylene, reactive adsorption, supported sulfonic acid

## Abstract

It is important to develop effective strategies for enhancing the removal capacity of aromatic volatile organic compounds (VOCs) by modifying conventional porous adsorbents. In this study, a novel HZSM-5 zeolite-supported sulfonic acid (ZSM−OSO_3_H) was prepared through ClSO_3_H modification in dichloromethane and employed for the elimination of gaseous *o*-xylene. The ClSO_3_H modification enables the bonding of −OSO_3_H groups onto the HZSM-5 support, achieving a loading of 8.25 mmol·g^−1^ and leading to a degradation in both crystallinity and textural structure. Within an active temperature range of 110–145 °C, ZSM−OSO_3_H can efficiently remove *o*-xylene through a novel reactive adsorption mechanism, exhibiting a removal rate exceeding 98% and reaching a maximum breakthrough adsorption capacity of 264.7 mg. The adsorbed *o*-xylene derivative is identified as 3,4-dimethylbenzenesulfonic acid. ZSM−OSO_3_H demonstrates superior adsorption performance for *o*-xylene along with excellent recyclability. These findings suggest that ClSO_3_H sulfonation offers a promising approach for modifying various types of zeolites to enhance both the elimination and resource conversion of aromatic VOCs.

## 1. Introduction

The emission of various aromatic volatile organic compounds (VOCs) from industrial activities poses a significant threat to human health and the atmospheric environment due to their high toxicity and carcinogenic properties [1,2,3,4]. In order to address safety concerns and comply with more stringent regulations, various techniques have been developed for the removal of aromatic VOCs [5,6,7,8]. Among them, adsorption was widely employed as an independent purification or pretreatment approach for capturing low-concentration aromatic VOCs (below 2500 mg·m^−3^) due to its operational simplicity and economic feasibility. However, the use of adsorbents with superior performance in capturing aromatic VOCs is a prerequisite [9,10,11].

A series of typical porous zeolites, such as ZSM-5, Y, X, and BEA, have been developed and applied for aromatic VOC adsorption [12,13]. Compared with commercial carbon-based materials such as activated carbons [14], activated carbon fibers [15], and biochar [16], zeolites exhibited superior regeneration performance, excellent thermal stability, and incombustibility [17]. More importantly, they also demonstrated a significant capacity or potential improvement in capturing aromatic VOCs. Furthermore, zeolites were crystalline aluminosilicate minerals characterized by a porous structure, and their adsorption performance is intricately linked to the distribution of pores and surface hydrophobicity. Therefore, optimizing pore distribution and surface modification are crucial strategies for effectively enhancing the adsorption capacity of aromatic VOCs [18,19,20,21].

The adsorption capacities and diffusion efficiencies of toluene were enhanced by the introduction of 5–10 nm mesopores on micro-porous ZSM-5 zeolite via NaOH etching [22]. The ZSM-5/SBA-15 composites were proven to have stronger hydrophobicity and higher toluene adsorption capacity compared to ZSM-5 due to the introduction of all-siliceous SBA-15 and the construction of intracrystalline mesopores [23]. In order to enhance the toluene adsorption capacity of zeolite under high relative humidity conditions, core-shell structured zeolite-hydrophobic organic polymer composites were synthesized [24]. However, previous studies have primarily focused on regulating the physical adsorption between zeolite and aromatic VOCs. Therefore, it is necessary to explore the selection of appropriate reagents to facilitate stronger chemical adsorption of aromatic VOCs on the zeolite by modifying its surface.

**Scheme 1:**
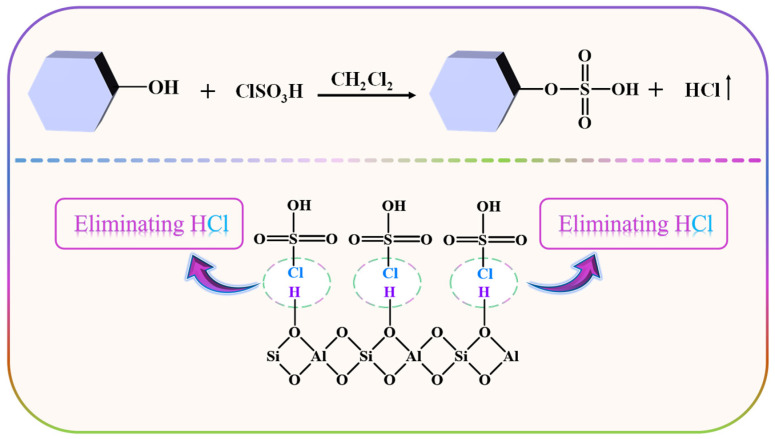
Schematic of the preparation for ZSM−OSO_3_H.

Herein, chlorosulfonic acid (ClSO_3_H) was chosen to modify HZSM-5 (proton form zeolite Socony Mobil-5), namely the HZSM-5 zeolite-supported sulfonic acid (ZSM−OSO_3_H) was synthesized by conducting a sulfonation reaction between ClSO_3_H and the ample hydroxy groups present in HZSM-5 (Scheme 1). Then, the enhanced removal ability of ZSM−OSO_3_H under different process conditions was examined using *o*-xylene as a representative pollutant of aromatic VOCs. Various characterization methods were employed to analyze the ZSM−OSO_3_H and the adsorbed *o*-xylene derivative on its surface. This study presents an investigation into the feasibility of utilizing ZSM−OSO_3_H as a heterogeneous reactive adsorbent for the removal and recovery of *o*-xylene from gas streams, thereby offering a potential approach to modify other types of zeolites in order to enhance the elimination of VOCs.

## 2. Results and Discussion

### 2.1. Structure and Basic Properties of Adsorbents

**Figure 1:**
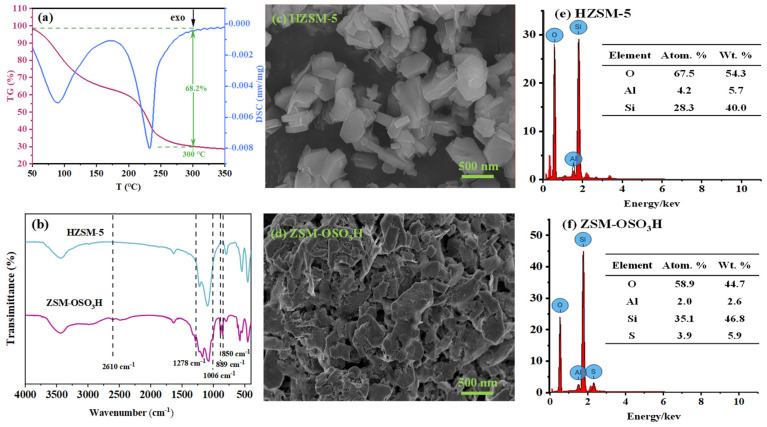
(**a**) TG and DSC curves of ZSM−OSO_3_H. (**b**) FT-IR spectrums of HZSM-5 and ZSM−OSO_3_H. SEM images and corresponding EDS spectra of HZSM-5 (**c**,**e**), and ZSM−OSO_3_H (**d**,**f**).

From Scheme 1, it can be observed that the sulfonic acid group of ZSM−OSO_3_H is anchored onto HZSM-5 through the sulfonation reaction of chlorosulfonic acid with its surface hydroxyl groups (eliminating HCl). After conducting an acid-base titration measurement, it was determined that the sulfonic acid loading was 8.25 mmol·g^−1^, from which its mass percentage content was calculated to be 66.8%. This value closely aligns with the weight loss rate (68.2%) of ZSM−OSO_3_H within the testing temperature range of 50–300 °C (Figure 1a) [25,26]. This suggests that the sulfonic acid groups were anchored on the HZSM-5, thereby improving its stability and reaction activity [26]. The ZSM−OSO_3_H FTIR spectrum exhibited additional bands at 2610, 1278, 1006, 889, and 850 cm^−1^ compared to the HZSM-5 spectrum (Figure 1b), which were attributed to the presence of anchored sulfonic acid [26,27]. The SEM images of HZSM-5 (Figure 1c) and ZSM−OSO_3_H (Figure 1d) revealed a dispersed polyhedral morphology of the particles. The ClSO_3_H modification induced an increase in surface roughness and aggregation tendency for ZSM−OSO_3_H, which could be attributed to the etching effect of ClSO_3_H in CH_2_Cl_2_ medium and the strong polarity (hydrophilicity) of anchored sulfonic acid groups. The etching and modification effects could be further confirmed through the EDS results (Figure 1e,f). In ZSM−OSO_3_H, the presence of sulfur indicated successful anchoring of sulfonic acid groups. Simultaneously, compared to HZSM-5, there was an increase in silicon content (from 28.3 atom.% to 35.1 atom.%) and a decrease in aluminum content (from 4.2 atom.% to 2.0 atom.%), which could be attributed to the minor leaching of Al^3+^ ions from the zeolite skeleton.

**Figure 2:**
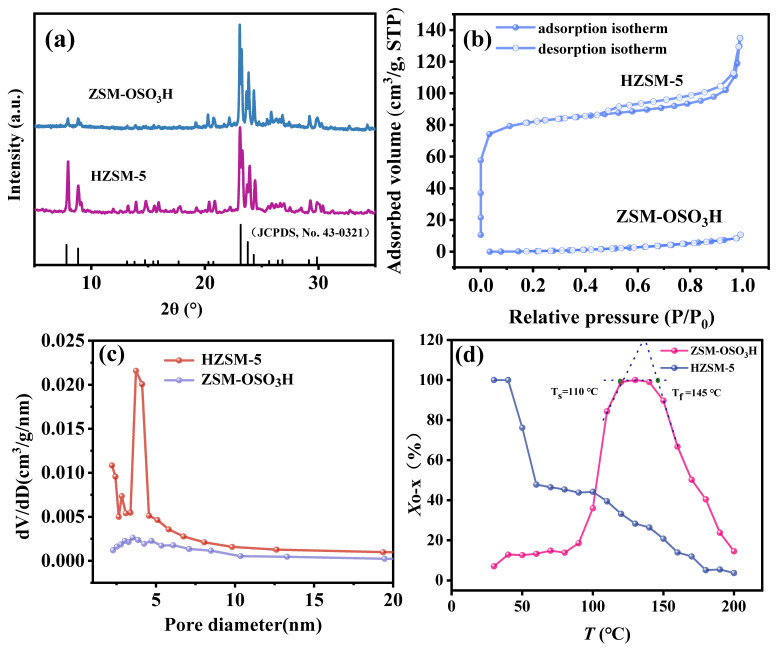
(**a**) XRD patterns of HZSM-5 and ZSM−OSO_3_H. (**b**) N_2_ adsorption/desorption isotherms of HZSM-5 and ZSM−OSO_3_H. (**c**) Pore size distribution of HZSM-5 and ZSM−OSO_3_H. (**d**) Relationship between *o*-xylene *X_o_*_-x_ and *T* for the HZSM-5 and ZSM−OSO_3_H.

The XRD patterns of HZSM-5 and ZSM−OSO_3_H adsorbents are illustrated in Figure 2a. The detected peaks at 2θ = 7.84, 8.73, 13.2, 13.9, 14.8, 15.5, 17.8, 20.8, 23.0, 23.8, 25.9, 26.8, and 29.9° corresponded to the (101), (200), (002), (102), (301), (302), (400), (103), (501), (303), (403), (104), and (503) planes, respectively, confirming the presence of highly crystalline MFI−structures in HZSM-5 [28,29]. The diffraction peaks in the 7–10° 2θ range of ZSM−OSO_3_H exhibited a significant attenuation, while those in the 13–18° 2θ range were nearly eliminated, which could be primarily attributed to the leaching of Al^3+^ ions induced by chlorosulfonic acid etching. Additionally, the etching effect of chlorosulfonic acid also results in the deterioration of the pore structure of ZSM−OSO_3_H. The N_2_ adsorption–desorption isotherms and pore size distribution curves are presented in Figure 2b,c, respectively. The HZSM-5 support exhibited appreciable adsorption at low relative pressures (*p*/*p*_0_ < 0.05), characterized by a prolonged horizontal plateau that eventually reached saturation due to the filling of micropores, indicating its high specific surface area (256 m^2^·g^−1^), pore volume (0.21 cm^3^·g^−1^), and narrow pore width (1.6 nm). However, the N_2_ adsorption–desorption isotherm of ZSM−OSO_3_H exhibited a type V isotherm (according to the six standard physisorption isotherms proposed by IUPAC). Additionally, its pore size distribution curve demonstrates an extremely low volume distribution (dv/dD), indicating an exceptionally low specific surface area (10 m^2^·g^−1^) and negligible pore volume (0.02 cm^3^·g^−1^), which might be attributed to the presence of anchored sulfonic acid group in the ZSM−OSO_3_H. These groups occupy the pore surfaces, resulting in blockage of the pore surfaces [25,26]. Considering that zeolite’s microporous network is composed of intersecting straight and sinusoidal channels (0.53 × 0.56 nm^2^ and 0.51 × 0.55 nm^2^, respectively) [30], the selective anchoring of sulfonic acid groups in specific pores may significantly impact the adsorption and diffusion performance of *o*-xylene, implying that regulating the porosity of zeolite could be an effective strategy to improve the distribution and quantity of anchored sulfonic acid groups.

### 2.2. Enhanced Removal Performance of o-Xylene on ZSM−OSO_3_H

The elimination performances of HZSM-5 and ZSM−OSO_3_H adsorbents were assessed through a series of *o*-xylene dynamic breakthrough tests conducted under various process conditions. The removal ratios of *o*-xylene were investigated as a function of temperature, ranging from 30 °C to 200 °C, using a continuous step−by−step heating manner (at a rate of 2 °C·min^−1^). The experiments were conducted with a bed height of 15 mm, a flow rate of 0.050 L·min^−1^, and an inlet concentration of 7.5 mg·L^−1^ (Figure 2d). The removal ratio of HZSM-5, like conventional porous adsorbent, exhibited a significant decline with increasing temperature, while maintaining the higher removal rate (>75%) at lower temperatures (<50 °C) [31,32,33]. This behavior could be attributed to the physical adsorption phenomenon resulting from its extensive surface area and pore volume [31,33]. The elimination of ZSM−OSO_3_H for *o*-xylene, however, presented an atypical behavior. Specifically, the removal curve of *o*-xylene could be divided into three distinct regions: the activation region (<110 °C), the active region (110~145 °C), and the deactivation region (>145 °C). The removal efficiency of *o*-xylene by ZSM−OSO_3_H increased with the rising temperature in the activation region and decreased in the deactivation region. In the active region, a stable elimination of over 98% of *o*-xylene from the gaseous stream was observed, indicating a robust interaction between the anchored sulfonic acid group of ZSM−OSO_3_H and *o*-xylene within this temperature range, thereby enhancing its elimination effectiveness. The reaction at the gas–solid interface resulted in the removal of *o*-xylene (refer to Section 2.3 for details). Therefore, this process can be referred to as reactive adsorption.

**Figure 3:**
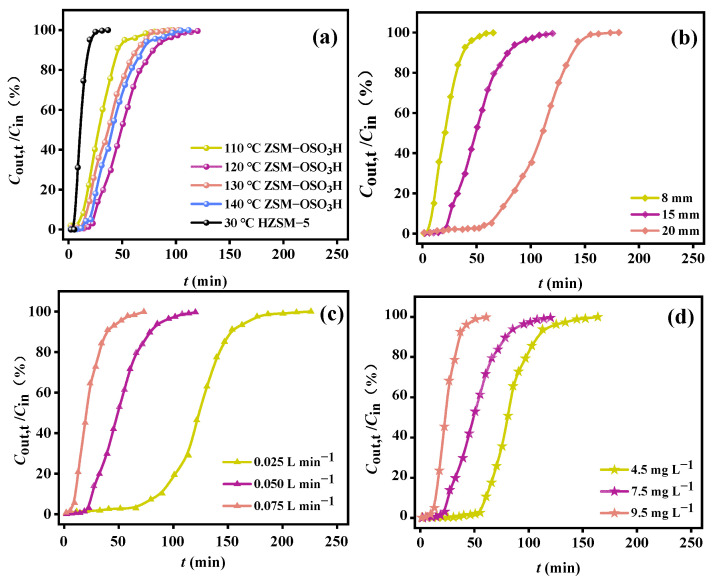
Adsorption breakthrough curves when separately varying (**a**) the adsorption temperature (*m* = 50 mg, *C*_in_ = 7.5 mg·L^−1^, *V*_g_ = 0.050 L·min^−1^), (**b**) the bed height (*C*_in_ = 7.5 mg·L^−1^, *V*_g_ = 0.050 L·min^−1^), (**c**) the gas flow rate (*C*_in_ = 7.5 mg·L^−1^, m = 50 mg), and (**d**) the inlet concentration (*V*_g_ = 0.050 L·min^−1^, m = 50 mg).

Inside the active region, four discrete temperatures (110 °C, 120 °C, 130 °C, and 140 °C) were selected to experimentally record the isothermal breakthrough curves of ZSM−OSO_3_H for *o*-xylene under identical process conditions (a bed height of 15 mm, a flow rate of 0.050 L·min^−1^, and an inlet concentration of 7.5 mg·L^−1^). The corresponding adsorption performance metrics are provided in detail in Table 1. For comparison purposes, the physical adsorption results of HZSM-5 for *o*-xylene at a temperature of 30 °C are also presented in Figure 3a. Clearly, the adsorption performance of ZSM−OSO_3_H was significantly superior to that of HZSM-5, particularly at the intermediate temperature of 120 °C. In comparison to HZSM-5’s physical adsorption of *o*-xylene, at this optimized temperature, ZSM−OSO_3_H’ *t*_B_ extended by 18.4 min, *Q*_B_ increased by 3.7 times, and *Q*_m_ increased by 3.4 times. The removal efficiency of ZSM−OSO_3_H towards *o*-xylene initially increased and then decreased as the bed temperature rose within a specific activation temperature range. This temperature−responsive behavior differed from that of aromatic VOCs by conventional porous materials, which exhibited a gradual decline in performance with increasing bed temperature [34,35,36,37]. However, while maintaining the bed temperature at 120 °C, variations in bed height, flow rate, and inlet concentration individually exhibited similar tendencies in terms of their impact on the removal efficiency of *o*-xylene as observed in conventional physical adsorption processes [35,36,37,38]. Specifically, the increase in bed height (Figure 3b), coupled with the decrease in flow velocity (Figure 3c) and initial concentration (Figure 3d), led to an upward trend for both *Q*_B_ and *Q*_m_ values. Under the conditions with flow rate of 0.025 L·min^−1^, initial concentration of 7.5 mg·L^−1^, and bed height of 15 mm, the maximum *Q*_B_ and *Q*_m_ were determined to be 264.7 mg·g^−1^ and 456.4 mg·g^−1^, respectively, showcasing the superiority of ZSM−OSO_3_H when compared with the physically adsorbing capacities of aromatic VOCs by common porous materials (Table 2). The enhancement effect might be attributed to the alterations in the interaction mechanism between *o*-xylene and ZSM−OSO_3_H induced by chlorosulfonic acid modification, rather than conventional enhancement of interface mass transfer.

### 2.3. o-Xylene Reactive Adsorption and Reusability of ZSM−OSO_3_H

**Scheme 2:**
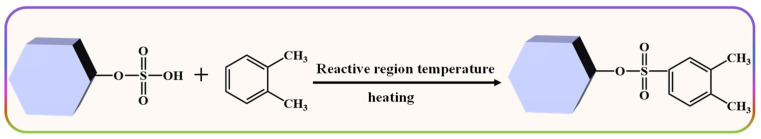
Schematic diagram and adsorption mechanism of the reactive adsorption process for removing *o*-xylene in ZSM−OSO_3_H.

**Figure 4:**
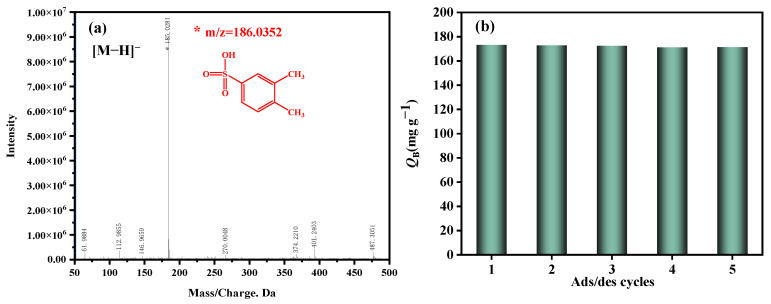
(**a**) ESI-MS spectra of adsorption product. (**b**) Reusability of ZSM−OSO_3_H in the repeated adsorption/desorption cycles.

The ethanol solvent was utilized to extract the adsorbed *o*-xylene derivative from the spent ZSM−OSO_3_H, followed by identification of the extracted derivative using the HPLC-MS methodology. The mass spectrum is presented in Figure 4a. In the negative ion mode, a prominent molecular ion peak at *m*/*z* = 185 was observed, indicating that the relative molecular mass of the adsorption product was 186. Therefore, a plausible mechanism for *o*-xylene elimination by the novel ZSM−OSO_3_H adsorbent has been proposed in combination with our previous research [25,26,44] (Scheme 2). The enhanced adsorption performance of *o*-xylene is achieved by undergoing a sulfonation reaction with the sulfonic acid group in ZSM−OSO_3_H, leading to its conversion into a non-volatile sulfonic acid derivative (*m*/*z* = 185), thereby facilitating its efficient removal from the gaseous stream. In this process, the sulfonic acid groups serve as active sites for the elimination of *o*-xylene, contrasting with the hydroxyl groups in unmodified HZSM-5 that function as ordinary adsorption sites. The sulfonic acid groups play a crucial role in this context. We propose that the surface reaction enthalpy between *o*-xylene and anchored sulfonic acid groups is more significant compared to its adsorption enthalpy with surface hydroxyl groups [45]. Obtaining relevant enthalpy data through DFT calculations, molecular modeling, or experimental studies will contribute to a deeper understanding of the adsorption process in perspective studies [46,47,48]. Additionally, the sulfonation reaction primarily involved electrophilic substitution, wherein electrophiles typically attacked the ortho- or para-position of the methyl group on the benzene ring due to their higher electron cloud density, rendering them more susceptible to electrophilic attack [49,50]. Consequently, it could be inferred that the resulting sulfonic acid derivative was 3,4-dimethylbenzenesulfonic acid.

After recovering the resulting 3,4-dimethylbenzenesulfonic acid from the spent ZSM−OSO_3_H through ethanol extraction, the HZSM-5 support was reclaimed. By subjecting it to chlorosulfonic acid modification once again in dichloromethane (3 g/15 mL) under ambient temperature, a new ZSM−OSO_3_H adsorbent was obtained and applied for subsequent reactive adsorption of *o*-xylene. After undergoing four cycles of adsorption, extraction, and re-sulfonation, Figure 4b illustrates the *Q*_B_ values of *o*-xylene for each treatment. The *Q*_B_ values for each ZSM−OSO_3_H adsorbent showed only minimal fluctuations between 171.2 mg·g^−1^ and 173.3 mg·g^−1^ compared to their initial use, thereby demonstrating the exceptional regeneration performance of the HZSM-5 support. To be more precise, the resulting adsorbed aryl sulfonic acid product could be recovered through solvent extraction, while the regenerated HZSM-5 zeolite could be reused for preparing a fresh batch of ZSM−OSO_3_H adsorbent, highlighting the advantages of this method in terms of both sorption efficiency and adherence to green chemistry principles.

## 3. Materials and Methods

### 3.1. Materials and Chemicals

The HZSM-5 zeolite with a SiO_2_/Al_2_O_3_ ratio of 46 was purchased from Nankai University Catalyst Co., Ltd. (Tianjin, China). All chemicals, such as chlorosulfonic acid, *o*-xylene, dichloromethane, potassium hydrogen phthalate, methanol, sodium hydroxide, and hydrochloric acid, were of analytical grade. The sulfonic acid group was simply incorporated into the HZSM-5 zeolite during the synthesis of ZSM−OSO_3_H. Specifically, the dichloromethane solution (10 mL) of chlorosulfonic acid (2.5 mL) was slowly added to a suspension of HZSM-5 in dichloromethane (3 g/15 mL) at ambient temperature and stirred for 3 h followed by alternating treatment of filtration and chloroform washing to give the sulfonated zeolite. The sulfonic acid loading of the ZSM−OSO_3_H was determined to be 8.25 mmol·g^−1^ using a classic acid-base titration method.

### 3.2. Adsorption Measurements

A custom-designed dynamic breakthrough test was developed to assess the performance of ZSM−OSO_3_H in removing *o*-xylene from gas streams. Prior to each test, the ZSM−OSO_3_H material was packed into a glass column with a diameter of 6 mm (φ6 mm), and the inlet concentration of *o*-xylene was adjusted to 7.5 mg·L^−1^ (*C*_0_). The flow rate was controlled at 0.05 L·min^−1^ using a flow meter, and the concentration of *o*-xylene was monitored using the flame ionization detector (FID) of a gas chromatograph (GC7900). The temperature range for the reaction between ZSM−OSO_3_H and *o*-xylene was determined through continuous step-by-step heating. The impact of typical process conditions, such as adsorption temperature, bed height, flow rate, and inlet concentration, on the adsorption capacity of ZSM−OSO_3_H for *o*-xylene was investigated through a breakthrough test. The removal ratios of *o*-xylene (*X_o_*_-x_, %) and the breakthrough adsorption capacity (*Q*_B_, mg·g^−1^) were calculated using Equations (1) and (2), respectively.

(1) $$X_{o\text{-}x}=\frac{{C_{\mathrm{in}}-C}_{\mathrm{out},t}}{C_{\mathrm{in}}}\times 100\%$$

(2) $$Q_{B}=\frac{V_{g}C_{\mathrm{in}}}{m}\int_{0}^{t_{B}}(1-\frac{C_{\mathrm{out},t}}{C_{\mathrm{in}}})\mathrm{dt}$$

where *V*_q_ (m^3^·min^−1^) represents the gas flow rate; m (g) is the adsorbent mass; *C*_in_ and *C*_out_ (mg·L^−1^) denote the inlet and outlet *o*-xylene concentrations in the gas flow, respectively; *t*_B_ (min) infers the breakthrough time, which is the time for the *C*_out_/*C*_in_ ratio to reach 0.05.

To identify the adsorbed *o*-xylene derivative and reusability of the spent ZSM−OSO_3_H, it was repeatedly extracted with absolute ethanol until aliquots of the extracts spotted onto thin layer chromatography plates were no longer visualized under UV light [44]. The residual solid was washed with distilled water and dried at 100 °C to recover HZSM-5 zeolite, and then the recovered HZSM-5 zeolite was converted again in dichloromethane (3 g/15 mL) under ambient temperature to the ZSM−OSO_3_H. The adsorbate was obtained by evaporating the extract [25,26].

### 3.3. Characterization

The morphology and chemical composition of the adsorbents were investigated using an S-4800 cold-field emission scanning electron microscope equipped with an INCA 350 energy dispersive spectrometer accessory (SEM-EDS; Hitachi, Tokyo, Japan). Fourier−transform infrared spectra (FTIR; KBr pellets) were recorded on a TENSOR 27 spectrometer (Bruker AXS, Karlsruhe, Germany). The N_2_ adsorption/desorption isotherms of the materials were measured at 77 K using a Kubo X1000 automatic surface area and pore analyzer (Beijing Builder Co., Ltd., Beijing, China). Prior to testing, the materials were subjected to treatment at 100 °C for 1.5 h in an MD-300 preprocessor (Beijing Builder Co., Ltd., China). The specific surface area, pore volume, pore width, and pore size distribution were determined using the Brunauer-Emmett-Teller (BET) and Barrett-Joyner-Halenda (BJH) models. The thermogravimetric (TG) and differential scanning calorimetry (DSC) analyses were conducted using the STA 449F 3TG/DSC analyzer (Netzsch, Germany). In this analysis, the samples were heated at a rate of 2.5 °C·min^−1^ from 50 to 300 °C under a nitrogen atmosphere. X-ray photoelectron spectroscopy (XPS) analysis was performed using an ESCALAB 250Xi spectrometer (Thermo Fisher, Dartford, UK) with monochromatic Al Kα radiation (hν = 1486.6 eV). The X-ray diffraction (XRD) analysis of the adsorbent was carried out using a D8 Advance X-ray diffractometer operating with Cu Kα radiation (λ = 0.154 nm; Bruker AXS, Germany). The adsorbed *o*-xylene derivative was subjected to analysis using high-performance liquid chromatography−mass spectrometry (HPLC-MS) on an Agilent 1260 system (Agilent Technologies, Santa Clara, CA, USA).

## 4. Conclusions

The HZSM-5 zeolite-supported sulfonic acid (ZSM−OSO_3_H), with a loading of 8.25 mmol·g^−1^, was synthesized via the ClSO_3_H sulfonation method in dichloromethane. The results obtained from TG/DSC, FTIR, SEM, XRD, and N_2_ adsorption–desorption analyses confirmed the successful bonding of sulfonic acid groups onto the HZSM-5 support. However, it was found that the modification with ClSO_3_H led to a degradation in both crystallinity and texture structure. The dynamic breakthrough test results demonstrated that the ZSM−OSO_3_H adsorbent exhibited an active temperature range of 110–145 °C, effectively eliminating over 98% of *o*-xylene from the gaseous stream. The removal efficiency of ZSM−OSO_3_H towards *o*-xylene initially increased and then decreased as the bed temperature rose within this active temperature range, showcasing its superior adsorption performance compared to HZSM-5. Furthermore, increasing the bed height while decreasing flow velocity and initial concentration led to a simultaneous increase in both *Q*_B_ and *Q*_m_ values. Under the given conditions with a flow rate of 0.025 L·min^−1^, initial concentration of 7.5 mg·L^−1^, and bed height of 15 mm, the maximum *Q*_B_ and *Q*_m_ values were determined to be 264.7 mg·g^−1^ and 456.4 mg·g^−1^, respectively. The HPLC-MS characterization revealed that the adsorbed *o*-xylene derivative was identified as 3,4-dimethylbenzenesulfonic acid. A plausible mechanism for *o*-xylene elimination by the novel ZSM−OSO_3_H adsorbent was proposed, i.e., the enhanced elimination of *o*-xylene was attributed to the sulfonation reaction of it with the sulfonic acid group in ZSM−OSO_3_H. Furthermore, the ZSM−OSO_3_H adsorbent demonstrated excellent recyclability and reusability over four consecutive cycles of adsorption/extraction/re-sulfonation. Therefore, ClSO_3_H sulfonation offers a promising approach for modifying various types of zeolites to enhance the elimination of VOCs.

## Figures and Tables

**Table 1:** Adsorption parameters for *o*-xylene under different process conditions.

		*t*_B_ (min)	*Q*_B_ (mg g^−1^)	*t*_m_ (min)	*Q*_m_ (mg g^−1^)
*T*	110 °C ZSM−OSO_3_H	11.2	81.8	79.9	224.1
	120 °C ZSM−OSO_3_H	23.3	173.3	114.1	383.7
	130 °C ZSM−OSO_3_H	15.1	112.1	82.6	286.9
	140 °C ZSM−OSO_3_H	18.9	140.9	104.5	324.3
	30 °C HZSM-5	4.9	36.9	36.7	87.5
*h*	8 mm	7.3	109.2	58.8	329.9
	15 mm	23.3	173.3	114.1	383.7
	20 mm	61.3	225.0	155.2	398.3
*V*_g_	0.025 L min^−1^	72.1	264.7	212.3	456.4
	0.050 L min^−1^	23.3	173.3	114.1	383.7
	0.075 L min^−1^	8.8	97.4	72.8	260.8
*C*_in_	4.5 mg L^−1^	57.2	254.9	152.4	372.5
	7.5 mg L^−1^	23.3	173.3	114.1	383.7
	9.5 mg L^−1^	12.7	119.9	60.9	230.9

**Table 2:** Evaluation of the adsorption capacity of diverse materials to *o*-xylene.

Adsorbent	Adsorption Capacity/(mg·g^−1^)	Reference
Bentonite	44.5	[39]
Alumina (γ-Al_2_O_3_)	99.8	[39]
GAC/NA	187.0	[40]
Carbon−based monoliths	58.3	[41]
Granular activated carbon	90.4	[42]
13X zeolite	17.7	[43]
Silica gel	96.5	[43]
Activated carbon	111.5	[43]
ZSM−OSO_3_H	456.4	Present work

## Data Availability

Data were contained within the article. The data presented in this study are available.
